# Medical Association of Central New York

**Published:** 1902-12

**Authors:** C. A. Greenleaf

**Affiliations:** Rochester, N. Y.


### SOCIETY PROCEEDINGS.

### Medical Association of Central New York.

REPORTED BY C. A. GREENLEAF, M. D., Secretary, Rochester, N. Y.

THE thirty-fifth annual meeting of the Medical Association of Central New York was held in the council chamber of the City hall, at Syracuse, October 21, 1902, at 10 o'clock a.m.

Dr. A. L. BEAHAN, president of the Association, called the meeting to order, at 10.30 a.m. He then stated that the first business in order would be the report of the secretary,

Dr. C. A. GREENLEAF, secretary—Inasmuch as this report has been printed in the Transactions, and as it has been customary in the past to omit it, for that reason, I move that the secretary's report be omitted.

Motion seconded and carried.

The president then announced the standing committees:

*Business Committee.*—S. F. Snow, Syracuse; W. T. Mulligan, Rochester; C. E. Congdon, Buffalo.

*Committee on Entertainment.*—John L. Heffron, E. J. Wynkoop, H. C. Baum, Syracuse.

*Committee on Credentials.*—A. L. Benedict, Buffalo; H. L. Elsner, Syracuse; Wm. Clapper, Victor.

*Committee on Publication.*—Wm. C. Krauss, Buffalo; C. A. Greenleaf, Rochester; Wm. M. Brown, Rochester.

The report of the treasurer, Dr. Wm. M. Brown, was then read and ordered printed:

| | |
|---|---|
| Dr. | |
| Cash from former treasurer | $229.48 |
| Back dues collected | 132.00 |
| | 361.48 |
| Cr. | |
| Disbursements | 162.21 |
| Balance on hand | $199.27 |

The president's address, entitled, The Surgical Specialty, was then read. (See page 313.)

Dr. S. F. Snow.—I move that a committee be appointed by the president to prepare resolutions on the death of Dr. E. M. Moore, and report at this afternoon's session.

Motion seconded and carried.

President Beahan.—I will appoint on this committee Dr. H. D. Didama, of Syracuse, chairman; Dr. A. C. Mercer, Syracuse, and Dr. C. A. Greenleaf, of Rochester.

The following papers were then read and discussed:

Practical diagnosis of gastric cancer, A. L. Benedict. Buffalo.

Salient points in the treatment of catarrhal deafness, S. F. Snow, Syracuse.

Two unusual cases of traumatism, W. C. Krauss, Buffalo.

Puerperal eclampsia, Wesley T. Mulligan, Rochester.

Fibroids or myomata with five cases with hysteromyomectomy and exhibition of pathological specimens, J. F. Myers, Sodus.

Some observations on the climate of the west, John L. Heffron, Syracuse.

The association then adjourned for lunch, tendered by the Onondago County Medical Society.

Meeting called to order for the afternoon session at 3 o'clock.

Dr. Nathan Jacobson, of Syracuse, then read a paper on Hemorrhagic appendicitis as the first manifestation of puerpera hemorrhagica.

Dr. Floyd S. Crego presented a report of a Case of myxedema with acromegaly.

The association then took up the matter of unfinished business.

Dr. C. A. Van der Beek.—I have two resolutions I wish to offer. The secretary of this organisation is accumulating quite a number of Transactions from year to year and they take up a great deal of his added space which he likes to devote to something else. I therefore move that these extra copies of Transactions be given to the Rochester Academy of Medicine for exchange.

Dr. Van der Beek.—Last year a resolution was presented before this society in Buffalo, which was laid upon the table at the suggestion of Dr. Howe, who was then president, and I have been requested to present it again. It is in regard to the establishment of a department for psychological research at Washington.

The committee on credentials recommended that the following delegates be elected to permanent membership in the association: Cyril Fulton, Lyons; J. S. Brand, Ontario County; C. C. Frederick, Buffalo; Milton E. Geery, Syracuse; E. P. Lothrop, Buffalo; C. E. Coon, Syracuse. Carried.

The election of officers for the years 1902-1903, resulted as follows: president, E. L. Heffron, Syracuse; first vice-president, C. A. Van der Beek, Rochester; second vice-president, M. L. Bennett, Watkins.

The secretary and treasurer were elected at the last meeting.

On invitation of Dr. J. P. Creveling, the association voted to hold its next annual meeting at Auburn. The Committee on Resolution made the following report:

Dr. Edward Mott Moore, of Rochester, well known here and abroad and universally respected, has passed away since the last meeting.

As he was the first president of this association it is especially appropriate that we should express our regret for his passing away and our sympathy with the afflicted family and all who were acquainted with him and knew his worth and worthiness.

As a husband, a father, a citizen, and a member of the medical profession he had few if any superiors.

We revere his memory and believe that it will be well for us to follow in his footsteps.

H. D. Didama,
Alfred Mercer,
C. A. Greenleaf.

The reading of papers was again in order, and the following were presented:

Some unusual causes of lead poisoning with report of a case, John Zimmer, Rochester.

The separation of the upper epiphysis of the humerus, D. M. Totman, Syracuse.

Surgical importance of the caput coli, and its etiologic factorate in producing inflammation of the vermiform appendix, J. P. Creveling.

Foreign bodies in the larynx and bronchi of small children, with report of six cases, J. H. Halstead, Syracuse.

Immediate repair of the soft parts following labor, Charles E. Congdon, Buffalo.

Utilisation of the sac in the radical operation for femoral hernia, A. L. Hall, Fulton.

The following papers were read by title:

Sympathetic ophthalmia (by invitation), George M. Case, Elmira.

The diagnosis of general paralysis of the insane, W. L. Russell, Willard.

Are the gonococci necessary as an infecting agent in inflammation of the female genitalia? J. Henry Dowd, Buffalo.

A vote of thanks was tendered the Onondaga County Medical Society for its kind hospitality, after which the society adjourned to meet at Auburn, on the third Tuesday of October, 1903.

A banquet was held at the Yates Hotel, at 7 o'clock in the evening, which was well attended and where the best of feeling was manifest.

